# Diabetic Phthisis and Its Treatment

**Published:** 1867-09

**Authors:** B. W. Richardson


### DIABETIC PHTHISIS AND ITS TREATMENT.

BY B. W. RICHARDSON, M.D.

The physician who has occasion to treat large numbers of patients suffering from phthisis pulmonalis soon becomes aware of the fact that the disease is not a single and simple organic lesion depending only on one primary condition, but that, manifesting a remarkable unity in respect to symtoms, it may exhibit as remarkable a diversity in relation to cause, and may be allied with many and varying forms of disease. Thus there is a true form of phthisis connected with alcoholic degeneration of the tissues—alcoholic phthisis; there is a form of phthisis in children connected with cyanosis—cyanotic phthisis; there is a bronchial phthisis; there is a form of phthisis from inhalation of foreign substances—mechanically excited phthisis; there is the ordinary phthisis of the young, hereditary or acquired; and, lastly, there is the peculiar form of phthisis which is sometimes coincident with diabetes, and to which I would now direct attention—diabetic phthisis.

Diabetic phthisis is, by comparison, a rare disease. In the course of twelve years' practice at the Royal Infirmary for Disease of the Chest I have met with not more than eight cases, and in private practice I have met with three only. On the whole, juding from experience, I should think that of fifteen hundred cases of a phthisical character, not more than one would be a case of diabetic phthisis. Further, the disease diabetes may prove fatal without any of the symptoms of phthisis. For all this, diabetic phthisis, when once set up, is a well marked form of disease: it is something more than a complication of diabetes when it appears, for it soon stands out first, and becomes the actual life-destroyer.

In three of my cases the patients presented themselves, unconscious that they were eliminating large quantities of sugar by the urine, and complaining solely of the affection of the chest. In all the other cases the symptoms of diabetes had been recognized prior to the development of pulmonary mischief; this, I have no doubt, is the general rule, and I believe in every case where the history of the symptoms can be clearly traced it will be found that the diabetes was the antecedent affection, although the patient was not conversant of the fact. When in the course of diabetes the phthisical condition is developed, the newly diseased state is not usually an early complication; but, once developed, it speedily runs its fatal course.

The first general symptom is severe hectic, the hot stage of which is very extreme, and, instead of being followed by profuse sweating, is succeeded by great coldness of the surface of the body, depression, and copious elimination of urine. Difficulty of breathing is a marked symptom; cough is common, but is usually hacking only, and is unattended with any quantity of expectoration. Hæmoptysis, in the strict sense of the word, I have not seen; but sputa of a rusty character, in small quantities, is frequent. There is little acute, thoracic pain, but great oppression. Waste of bodily substance is extreme.

The physical signs are well marked. If the disease is seen early, patches of lung, like so many centres, give signs of dry crepitation; rapidly this crepitation extends over the whole lung. In one of my cases, during the last six weeks of life I

could put the stethoscope over no part of the chest without hearing crepitation. The crepitation is distinctly that of early tubercle; it is wanting in the fineness of early pneumonic crepitation. In course of time there is some tendency to softening of tubercle, but this is very limited, and I have but once observed the actual formation of cavity. Death takes place, in fact, too early to give time to give time for softening or absorption of tubercle; added to this, the diabetic condition seems to interfere with the process of softening, probably by the removal of water from the tissues.

Percussion over the chest, where there is crepitation may be dull, but this sign is not essential.

Whenever there is clearly developed diabetic phthisis the prognosis is inevitably bad, according to our present knowledge of treatment. Further, the prognosis is almost definite as to time; I have not seen a case that survived four months after the tubercular condition had been obviously present. From six to ten weeks is the common duration of the term of life from the period of severe and definitely recurring hectic.

After death the condition of lung is peculiar, and in three cases—the only cases I could be allowed to inspect—the condition was the same. The lungs were much shrunken and dry; they were greyish and darkly mottled in color; the tissue was filled with small dark—I had almost said gritty—tubercle, and there were a few patches of deep vascular congestion. There was no pleuritic adhesion, no serious effusion, no pulmonary cavity.

In every case of diabetic phthisis I have seen there has been disease of the base of the brain. In one case there was a growth of bone pressing upon the under surface of the medulla oblongata; in another case there was softening of brain substance, and in a third case there was disease of the vessels with thickening of the membranes and old adhesions. In one of these cases the patient had been under the late Dr. Bayly for "acute meningitis," and his symptoms of diabetes followed that attack immediately. As he recovered from his acute illness he discovered himself diabetic.

The pathological relationship of diabetes and phthisis of the lung seems to me to be through the nervous system. That there is a functional and an organic type of diabetes; that the functional type is largely curable, and the organic absolutely incurable; and that the functional type is connected with a false digestion, owing to temporary interference with nerve action—these, I think, are facts which every scientific physician must be prepared to accept.

Some difference of opinion, however, yet exists as to the relationship of diabetes to disease of the brain. The progress of experimental inquiry has all been to the effect that lesion of brain structure is an efficient cause of the diabetic condition; but Dr. Ogle has recently, in a most labored and able paper, maintained that the brain lesion sometimes found in diabetes is a result of structural change incident to the diabetic state, and a result instead of a cause. I do not propose to discuss this refined question now, but I would point out that when phthisis of the lung is developed during diabetes, the morbid change appears to be the result of what may truly be called inervation of the lung tissue. That the change is not due merely to modification of the blood is certain from the fact that diabetes may exist or prove fatal without the occurrence of pulmonary phthisis or any sign of it. The occurrence of the phthisis also takes place, as I think experience shows, only when the diabetes depends on lesion at the base of the brain. It is fair to presume, therefore, that in such cases the nervous injury has so extended as to involve at their source the nerves from which the pulmonic structure is supplied.

Regarding treatment in diabetic phthisis, I have tried various plans—oxygen by inhalation, oxygen by per-oxide of hydrogen, special diet, change of air—and all to no purpose. Still there are certain points of practice which are worthy of note. I name two especially:

1. I am convinced that in this malady oxygen and its allies, chlorine, iodine, or their compounds, do harm: they increase elimination, and reduce accordingly.

2. I am equally certain that a diet restricted to albuminous products is utterly wrong, both in theory and in practice. I believe that in functional diabetes a great duty can be effected by restricted diet, coupled with the method first suggested by Rollo, of giving with such diet ammonia and iron freely. I doubt, however, the practice of restricted diet in every case of organic diabetes, and in cases where there is the faintest indication of phthisis the restricted diet becomes, I feel sure, a positive evil. So soon as diabetic phthisis is established, the general dietetic rules for phthisis alone are the rules, and the only rules, to follow. In the way of affirmative treatment the principles are—to sustain warmth of body, to check waste by opium and quinine, and to sustain by good food, especially by the free use of animal oil. In the next case I have to treat I will give animal oil, not by the spoonful, but by a half-pint at a time. I will give it as the Esquimaux takes it, and for the same reason, to sustain the lost caloric in his case, too rapidly carried away by the surrounding cold, and in the case of the diabetic man by the excessive formation, dissolution, and elimination of sugar. To this last remark I would add that, in respect to the treatment of organic diabetes altogether, there is more hope in the free use of animal oil than in any other remedy. Here is an unexplored field of practice before us, a very simple practice, and easily put to the test, but a practice strictly rational and based on the true institutes of medicine.—*Medical Times and Gazette.*

